# Developing a Beginner’s Guide to Writing a Clinical Case Report: A Pilot Evaluation by Junior Doctors

**DOI:** 10.7759/cureus.6370

**Published:** 2019-12-13

**Authors:** Samson O Oyibo

**Affiliations:** 1 Diabetes and Endocrinology, Peterborough City Hospital, Peterborough, GBR

**Keywords:** education, evaluation, case reports, medical writing, a guide, mentorship, junior doctors' education, teaching, online presentation, pilot study

## Abstract

Introduction

Writing a case report increases one’s knowledge about a particular disease condition, demonstrates intellectual curiosity and commitment to scientific inquiry and the ability to follow through on scholarly projects. Despite several articles and journal-specific instructions published concerning case report writing, none have been evaluated by their intended audience. The aim of this study was to get junior doctors to evaluate an online presentation as part of the process of developing a beginner’s guide to writing a clinical case report.

Materials and methods

In response to our previous studies an online presentation concerning how to write a clinical case report was provided for junior doctors. Junior doctors were invited by email to look at the online presentation and complete an online evaluation form thereafter. The questions were adapted from the Evaluation Form for Teaching and Presentations provided by the Joint Royal Colleges of Physicians Training Board. Data was analysed both quantitatively and qualitatively.

Results

Sixty-five doctors looked at the presentation and completed the online evaluation form. All agreed that the objectives of the presentation were identified and met. Sixty-four (98.5%) agreed that it was effective and clear. Sixty percent indicated that they found the information and instructions useful. An additional 13.85% found the whole presentation useful without specifying any aspect. Eight percent found the summary slide useful, 4.62% found the case selection criteria slide to be useful and 4.62% found the permission and patient consenting slide useful. Twenty percent would like the inclusion of examples of good abstracts and case reports, 13.85% would like more teaching sessions, and 13.85% would like improvements to the slide-presentation format. Overall, 64 junior doctors (98.46%) remarked that the presentation was good, very good or excellent.

Conclusions

This study has demonstrated the importance of evaluation of teaching material by junior doctors while developing a beginner’s guide to writing a clinical case report. Once the above action points and limitations have been taken into account, further repeat evaluations by junior doctors need to be undertaken while developing a robust beginner’s guide to writing a clinical case report.

## Introduction

Having an article published in a peer-reviewed medical journal is important for career progression in several medical specialties. Although enhancement of their curriculum vitae has been cited as a motivation to getting published, a keen interest in the subject is a more important reason stated by doctors [1]. Writing up a case report increases one’s knowledge about a particular disease condition, demonstrates intellectual curiosity and commitment to scientific inquiry and the ability to follow through on scholarly projects [2].

In a previous study, we demonstrated that junior doctors feel that medical article publishing is an effective teaching method but little was done to help them bridge the gap between getting an interesting case and getting published [3]. In a follow-up study, we highlighted the importance of establishing a medical article publishing club for junior doctors based on action points from the previous study. Junior doctors said that the medical article publishing club contributed to learning, education and publishing skills [4].

In response to action points from the above-mentioned studies an online PowerPoint presentation was provided for junior doctors on “a guide to writing a clinical case report”. The main objective of this study was to obtain junior doctors’ evaluation of the online presentation, with the ultimate aim of making improvements and developing a robust and user-friendly guide to writing clinical case reports.

## Materials and methods

The online presentation

**Figure 1:**
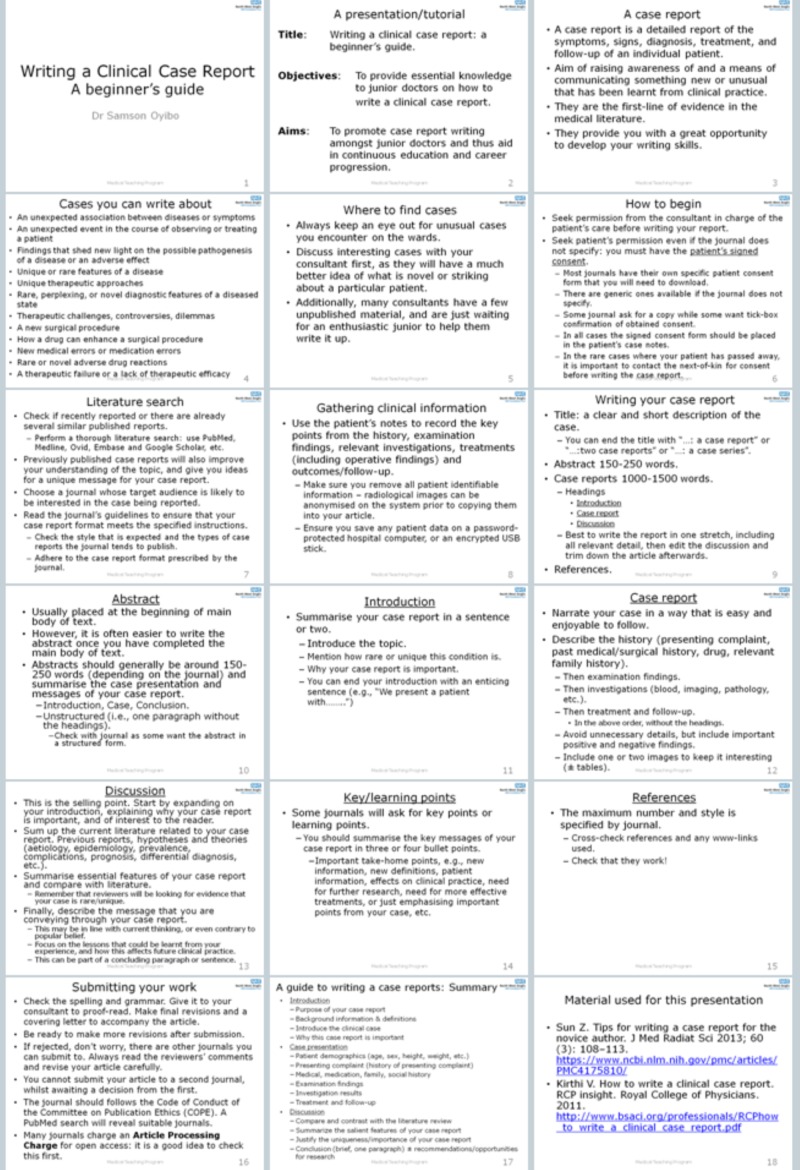
Copy of slides from PowerPoint presentation “A beginner's guide to writing a clinical case report”.

As an action point to a previous study an online PowerPoint presentation of “a guide to writing a clinical case report” was made for junior doctors to aid them in writing clinical case reports. This consisted of 18 PowerPoint slides starting from the title slide to the bibliography slide. This presentation was made available on our institution’s educational website for all junior doctors to use. The PowerPoint presentation is shown in Figure 1.

Study participants

Junior doctors in our healthcare institution were invited by email to look at the online PowerPoint presentation and complete an online evaluation form thereafter. There was also the facility to download the presentation. Invited doctors were given four weeks to respond while a reminder invitation email was sent every week for the same four-week period.

Study design

As part of the email, a web-based evaluation form was administered to junior doctors so that they could evaluate the online PowerPoint presentation after going through it. The evaluation form distribution and data collection were carried out over a four-week period. Ethics approval was sought through the Research & Development department of our institute. This study did not require ethical approval on the account of it being registered with our Quality, Governance and Compliance Department as a Quality of Education Improvement Project. Participants were assured of strict anonymity and confidentiality during this study.

Evaluation questionnaire

The evaluation questionnaire was prepared online using SurveyMonkey [5]. The questions were adapted from the Evaluation Form for Teaching and Presentations provided by the Joint Royal Colleges of Physicians Training Board [6]. The questionnaire contained six questions: (1) were the objectives of the online presentation identified, (2) were the objectives met, (3) was the delivery of the presentation effective and clear, (4) what aspects of the presentation was useful, (5) any suggestions for improvement, and (6) overall, what is your evaluation of the online presentation. Questions 1-3 required a “yes” or “no” answer. Questions 4-5 were open-ended questions requiring input into a comment box. Question 6 required an answer from “very bad”, “poor”, “fair”, “good”, “very good” or “excellent”. A web-link to the questionnaire was sent via email to participants.

Data analysis

The responses to questions 1, 2, 3, and 6 were analyzed and presented as whole numbers (and percentages). The answers to questions 4 and 5 were transcribed verbatim and analyzed qualitatively by the process of thematic analysis [7, 8]. The data was reviewed for initial codes, subthemes and subsequently developed themes related to what was found useful and suggestions for improvement. The raw data, subthemes and themes were continuously reflected upon to ensure credibility and trustworthiness of this survey [9].

## Results

There were 65 respondents to the invitation emails. Therefore, 65 junior doctors looked at the presentation and completed the online evaluation form.

Objectives, clearness and effectiveness

All 65 respondents (100%) agreed that the objectives of the presentation were identified. All 65 respondents (100%) agreed that the objectives of the presentation were met. Sixty-four respondents (98.5%) agreed that the presentation was effective and clear. This is shown in Table 1.

**Table 1:** Answers to questions 1-3 of the evaluation form filled out by the junior doctors.

Questions concerning the presentation	Number of junior doctors (N = 65)	
	YES	NO
Question 1: Were the objectives of the presentation identified?	65	0
Question 2: Were the objectives met?	65	0
Question 3: Was the delivery of the presentation effective and clear?	64	1

Useful aspects and suggestions for improvement

The answers to questions 4 and 5 were analysed thematically. The raw data (answers to both questions along with the thematic analysis) used to support the findings of this study has been deposited in the Harvard Dataverse and is freely accessible [10]. The main themes derived from the analysis are presented here.

Question 4 - What Aspect of the Presentation Was Useful?

All respondents answered question 4, and several major themes emerged from the thematic analysis. Thirty-nine respondents (60%) indicated that they found the information and instructions provided in the presentation useful (e.g., they highlighted the stepwise approach, breakdown, clear, concise and systematic structure of the information provided). Nine respondents (13.85%) indicated they found the whole presentation useful without specifying any aspect. Five respondents (7.69%) indicated that they found the summary slide useful. Three respondents (4.62%) indicated that they found the case selection criteria slide to be useful. A similar number of respondents (4.62%) indicated that they found the permission and patient consenting slide useful. One respondent particularly found the abstract slide useful. Two respondents indicated that the subject/topic was useful. Two respondents made an abbreviated text comments which could not be deciphered while one respondent indicated that the presentation was “a bit vague”.

Question 5 - Any Suggestions for Improvement?

Sixty-two respondents answered question 5, and several major themes emerged from the thematic analysis. Thirteen respondents (20%) indicated that they would like the inclusion of examples of good abstracts and case reports. Nine respondents (13.85%) indicated that they would like more presentations and teaching sessions (e.g., workshop sessions, online sessions and circulation of the presentation to more junior doctors and medical students). Nine respondents (13.85%) indicated that the slide-presentation format could be improved (e.g., add more colour, make the slides more interactive, less crowded, less rushed, shorter presentation). Thirty respondents (46.15%) indicated “nil” or “none” in response to the question “any suggestions for improvement”. Two respondents just gave praises (e.g., good job, well done), one respondent made an abbreviated text comment which could not be deciphered, and another left that question blank.

Overall evaluation of the presentation

Sixty-four respondents (98.46%) remarked that the presentation was good, very good or excellent. One respondent remarked that the presentation was poor. This is shown in Table 2.

**Table 2:** Junior doctors’ overall evaluation of the presentation.

Overall evaluation of presentation	Number of junior doctors (%)
Excellent	36 (55.38%)
Very good	20 (30.77%)
Good	8 (12.31%)
Fair	0
Poor	1 (1.54%)

## Discussion

Formal training and adequate mentorship are key ingredients required to help junior doctors with writing and presenting case reports. The importance of lack of these factors has been highlighted in a previous study looking at the perceptions of fourth-year medical students on writing case reports [11]. In this study, medical students indicated that lack of formal training and lack of mentorship were significant barriers to writing and presenting cases. There are several journal-specific guides and instructions on how to write clinical case reports but despite this, junior doctors still find it difficult to write up a case report. This fact emphasizes the importance of mentorship and training, which could be provided by a curriculum-based medical article publishing club or forum, which should include an easy-to-follow guide to writing case reports for junior doctors. While developing such a guide it is important that there is continuous evaluation by the junior doctors. Evaluation should be a continuous and periodic process, as it helps teachers and learners to improve the teacher-learner process.

There are several articles and journal-specific instructions published concerning writing clinical case reports but there is scarcity of reports of evaluation of these published guides and instructions by their intended audience. A guide to writing case reports directed at junior doctors in a user-friendly format and evaluated by junior doctors may go a long way in helping junior doctors write up clinical case reports. Such a guide can be included in the junior doctors’ teaching curriculum alongside an adequate mentorship program.

Action points from this pilot study

This study has demonstrated the importance of evaluation of teaching material by the intended learners, the junior doctors in this case. Junior doctors found the PowerPoint presentation about a “guide to writing a clinical case report” useful. In particular: the layout of the instructions, the information about permission and patient consenting, the information about case selection criteria, and the summary slide at the end of the presentation. The junior doctors also suggested ways of improving the presentation, namely, inclusion of examples and illustrations of good abstracts and case reports, adding colour to the presentation and making it more interactive and providing more teaching sessions and presentations on the topic of writing clinical case reports. These factors will be taken into account while making the improvements to this guide.

Limitations

This study has some limitations that should be acknowledged. First, this study assumes that everyone who looked at the presentation went on to complete the evaluation form. We have no way of knowing how many junior doctors looked at the presentation without going on to complete the online evaluation form. There are various forms of page-view/download counters that can be used to access this data when arranging future studies. Second, the results of this pilot study may not be generalizable as the sample size (respondents) makes up 25% of the total junior doctor population in just one healthcare institution. However, this was a pilot study. Third, the invited population of doctors are employees within the same healthcare establishment as the organiser of the study. Therefore, any non-responder or responder bias based on this cannot be ruled out. A sample size including junior doctors from different healthcare institutions would limit this bias.

## Conclusions

This study has demonstrated the importance of evaluation of teaching material by junior doctors while developing a beginner’s guide to writing a clinical case report. Once the above action points and limitations have been taken into account and improvements made, further repeat evaluations by junior doctors will need to be undertaken while developing a robust beginner’s guide to writing a clinical case report.
